# Impact of marital status on survival of gastric adenocarcinoma patients: Results from the Surveillance Epidemiology and End Results (SEER) Database

**DOI:** 10.1038/srep21098

**Published:** 2016-02-15

**Authors:** Miaozhen Qiu, Dajun Yang, Ruihua Xu

**Affiliations:** 1 Department of Medical Oncology, Sun Yat-Sen University Cancer Center; State Key Laboratory of Oncology in South China; Collaborative Innovation Center for Cancer Medicine, 651 Dongfeng Road East, Guangzhou 510060, China; 2 Department of Pathology, The Johns Hopkins University School of Medicine, Baltimore, MD 21231, USA; 3 Department of Experimental Research, Sun Yat-Sen University Cancer Center; State Key Laboratory of Oncology in South China; Collaborative Innovation Center for Cancer Medicine, 651 Dongfeng Road East, Guangzhou, China

## Abstract

Marital status was found to be an independent prognostic factor for survival in various cancer types. In this study, we used the Surveillance, Epidemiology and End Results database to analyze the survival difference among different marital status in the United States. Gastric adenocarcinoma patients from 2004–2012 were enrolled for study. The 5-year cause specific survival (CSS) was our primary endpoint. Totally 29,074 eligible patients were identified. We found that more male patients were married than female. Asian patients had the highest percentages of married than the other races. More married patients were covered by the insurance. Married patients had better 5-year CSS than unmarried, 30.6% vs 25.7%, P < 0.001. The median overall CSS was 17.87 and 13.61 months for the married and unmarried patients, hazard ratio: 1.09 (95% confidence interval: 1.01–1.17), P = 0.027. The survival difference was significant in the insured but not in the uninsured patients. Widowed patients had the worst prognosis compared with other groups even though they had more stage I disease and more well / moderate differentiated tumors. These results indicated that unmarried gastric adenocarcinoma patients were at greater risk of cancer specific mortality. We recommend every patient should have access to best available gastric cancer therapy.

Although the incidence and mortality has declined over the last half-century, gastric cancer remains the fourth most common cancer and the second most frequent cause of cancer related death worldwide^1^,^2^,^3^. According to the GLOBOCAN 2012, the estimate new cases of gastric cancer are 631,300 for male and 320,300 for female^4^. Adenocarcinoma is the most frequent histologic subtype of gastric cancer^5^. Despite the success of modern chemotherapy, the prognosis for gastric cancer patients is still dismal^6^. Finding out potential prognostic factors is helpful for us to set up individual therapy schedule and improve the survival.

Greater longevity of married people compared with unmarried persons has been demonstrated^7^,^8^,^9^. However the impact of marital status on disease specific survival among cancer patients has been controversial, including protective^7^,^10^,^11^,^12^,^13^,^14^,^15^, mixed^16^,^17^ and no effect^18^,^19^. A systematic review and meta-analysis demonstrated that all non-married conditions (widowed, divorced/ separated and never married) were associated with a significantly greater risk of death, as compared to married individuals^20^. In a Surveillance, Epidemiology and End Results (SEER) based study, Aizer AA *et al.* identified 1,260,898 patients diagnosed between 2004 and 2008 with lung, colorectal, breast, pancreatic, prostate, liver/intrahepatic bile duct, non-Hodgkin lymphoma, head / neck, ovarian and esophageal cancer for analysis^4^. They found that unmarried patients were at significantly higher risk of metastatic diseases, under treatment and cancer related death^10^. Zhou RP *et al.* using the SEER database to investigate the relationship between marital status and the survival of gastric cancer patients and they found that unmarried patients were at higher risk of cancer related death^15^. However, the authors mixed the carcinoid tumor/neuroendocrine, gastrointestinal stromal sarcoma and adenocarcinoma together. In the present study, we would like to focus on the gastric adenocarcinoma, which is the most common histology subtype for gastric malignance diseases.

## Results

### Patient baseline characteristics

The study identified 29,074 gastric adenocarcinoma patients. Of these patients, 18,284 (62.89%) were male and 10,790 (37.11%) were female. The median age of the whole group was 67 years old. Totally 17,854 (61.41%) patients were married and 11,220 (38.59%) were unmarried including 4,353 (14.97%) widowed, 4,139(14.24%) single and 2,728 (9.38%) separated / divorced. Table 1 showed the relationship between clinicopathologic features and marital status.

The ratio of male to female was highest in the married group (2.56) while it reversed in the widowed group (0.44). The mean age of patients in the widowed group was significantly higher than in other subgroups. Asian patients had higher percentage of married than other races, and African-American had the lowest. As for the insurance status, we found that more married patients were covered by the insurance and more single patients were uninsured.

Widowed patients had more antrum/pylorus and lesser/greater curvature cancer, more tumors at stage I and well/moderately differentiated tumors. Patients in the married group had more stage II/III diseases. Single patients had more stage IV diseases, more signet ring cell and poorly differentiated tumors.

There is no information of chemotherapy in the SEER database. We only collected the information of surgery and radiotherapy. The percentage of patients who received therapy (surgery or radiotherapy) from stage I to IV was 54.89%, 54.47%, 55.78% and 55.81%, P = 0.438. Similarly, no significant difference was found for patients with different histologic subtype to receive treatment, 55.48% for adenocarcinoma, 54.53% for mucinous adenocarcinoma and 55.02% for signet ring cell carcinoma, P = 0.663. More patients in the married group received surgery with or without radiation than those in the unmarried group.

### Survival

**Figure 1:**
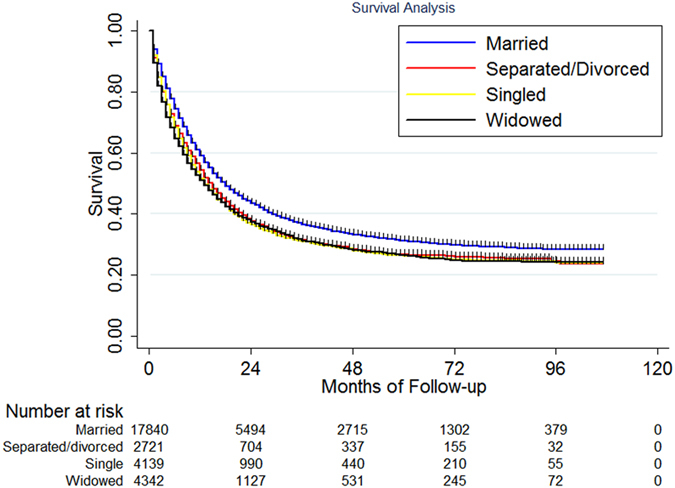
The survival difference among different marital status.

**Figure 2:**
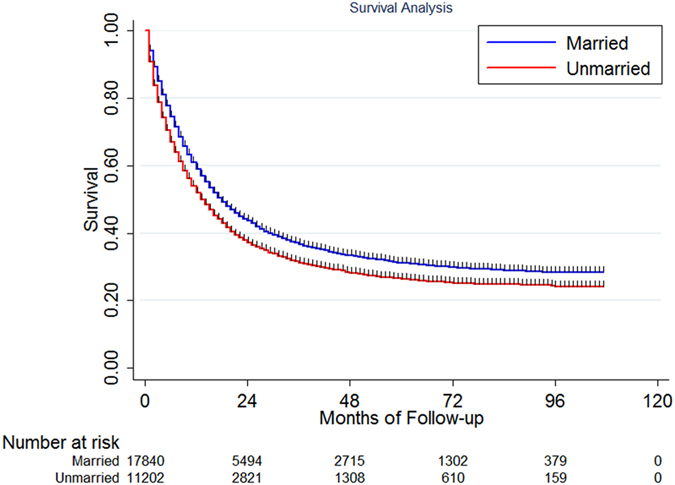
The survival difference between married and unmarried patients.

In this study, 17197 deaths (59.13%) were observed including 10289 (57.63%) in the married group (N = 17854), 2712 (62.30%) in the widowed group (N = 4353), 2522 (60.93%) in the single group and 1668 (61.14%) in the separated/divorced group. The median survival for the whole population was 16.08 months with a 5-year cause specific survival (CSS) of 28.7% [95% confidence interval (CI): 28.1–29.4%]. The median overall CSS was 17.87 and 13.61 months for the married and unmarried patients, P < 0.001. The 5-year CSS was higher in the married group than in the other groups, 30.6% in married group, 25.3% for the widowed group, 25.4% for the single group and 26.2% for the separated/divorced group, Fig. 1. Since the survival difference among patients in the widowed, single and separated/divorced groups was small, we combined these three groups into a new group called unmarried. The median survival for patients in unmarried group was 13.61 months and the 5-year CSS was 25.7% (95% CI: 24.7%–26.7%). The survival difference between married and unmarried group was significant, P < 0.001, Fig. 2.

Table 2 demonstrated the comparison of median survival and 5-year CSS in different variables. Compared with female patients, male patients had a slightly better survival. The 5-year CSS was 28.8% vs 28.5%, P = 0.0285. Patients with tumor in the lesser/greater curvature had the best survival with a 5-year CSS of 39.0%. Asian patients had a significantly better survival than patients in other race/ethnicity. The 5-year CSS was 40.1% in Asian patients, 26.7% in African-American patients and 26.6% in the Caucasian patients, P < 0.001. We also analyzed the influence of insurance on the survival and found that the 5-year CSS was 8.5% higher in the insured group than uninsured group, 29.9% vs 21.4%, P < 0.001. The 5-year CSS for patients from AJCC 6^th^ TNM stage I to IV patients was 61.9%, 40.9%, 23.3% and 4.8% respectively, P < 0.001. The median survival for patients in stage I has not yet reached. For patients who received resection, the number of lymph nodes resected also had an effect on the survival. Patients with the number of lymph nodes resected over 3 had a significantly better survival than those with 1–3 lymph nodes resected, 45.7% vs 39.5%, P < 0.001. The survival became poorer as the tumor grade progressed from well to undifferentiated, 58.7% for well differentiated, 39.4% for moderately differentiated, 24.9% for poorly differentiated and 22.4% for undifferentiated tumors, P < 0.001.

### Male versus Female

**Figure 3:**
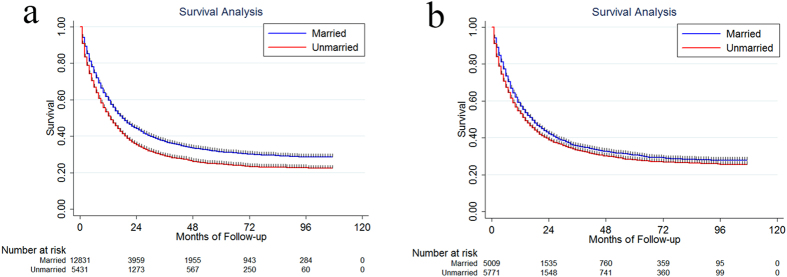
The survival difference between married and unmarried patients in male (**a**) and female (**b**).

Since some studies have identified a differential effect of marriage in men versus women, we also made a comparison of the prognostic effect of marital status between male and female. The median survival and 5-year CSS for married male were 18.22 months and 30.8% (95% CI: 29.8–31.8%). For unmarried male, they were 13.08 months and 24.0% (95% CI: 22.6–25.5%), P < 0.001 (Fig. 3a). For married female, the median survival and 5-year CSS were 17.13 months and 30.0% (95% CI: 28.5–31.5%). They were 14.11 months and 27.30% (95% CI: 25.9–28.7%) for unmarried female, P < 0.001 (Fig. 3b).

### Insurance and Race/Ethnicity

In order to find out potential reasons for the survival disparity between married and unmarried patients, we further explored the effect of insurance and race/ethnicity on the survival.

**Figure 4:**
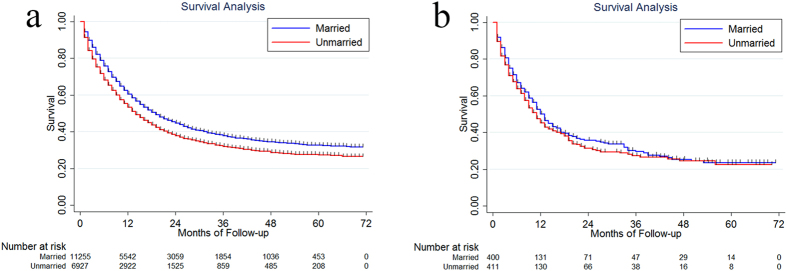
The survival difference between married and unmarried patients in insured (**a**) and uninsured patients (**b**).

For patients who were covered by the insurance, married patients had a significantly better median survival and 5-year CSS than unmarried patients, which were 18.07 months, 30.8% (95%CI: 29.5–32.1%) and 13.30 months, 24.8% (95% CI: 23.2–26.4%), P < 0.001 (Fig. 4a). While for those uninsured, the marital status had no effect on the survival, which were 11.99 months, 20.9% (95% CI: 15.6–26.7%) and 9.82 months, 18.5% (95% CI: 12.9–24.9%), P = 0.2627 (Fig. 4b).

**Figure 5:**
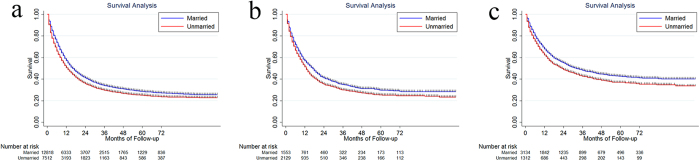
The survival difference between married and unmarried patients in Caucasian (**a**), African-American (**b**) and Asian (**c**).

The median survival and 5-year CSS for married Caucasian patients were 15.69 months and 27.2% (95% CI: 26.2–28.2%). For unmarried Caucasian patients, they were 12.20 months and 23.0% (95% CI: 21.7–24.3%), P < 0.001 (Fig. 5a). The median survival and 5-year CSS were 16.12 months, 28.7% (95% CI: 25.8–31.6%) for married African-American, and 12.19 months, 22.3% (95% CI: 20.0–24.7%) for unmarried African-American, P = 0.002 (Fig. 5b). The median survival and 5-year CSS were 28.23 months, 41.1% (95% CI: 37.9–42.3%) and 20.31 months, 32.3% (95% CI: 28.9–35.7%) for married and unmarried Asian patients, respectively, P = 0.0001 (Fig. 5c).

### Multivariate analysis

Variables showing a trend for association with survival (P < 0.05) were selected in the cox proportional hazards model. Sex, location and insurance status was not independent prognostic factors. Age, marital status, race/ethnicity, TNM stage, number of lymph nodes resected, histologic subtypes and grade were all independent prognostic factors in the multivariable analysis (Table 3). Compared for married patients, the HR for unmarried patients was 1.09 (95% CI: 1.01–1.17), P = 0.027.

## Discussion

We found that unmarried gastric adenocarcinoma patients (including widowed, single and separated/divorced), were at significantly greater risk of cause-specific death than married patients. After adjusting for demographics, stage and histologic subtypes, marital status remained independent prognostic factors. The insurance status reflected the socioeconomic status of patients and was reported to influent the overall survival of gastric cancer patients^21^. According to our result, more married patients were covered by insurance. Moreover, for insured patients, patients who were married had significantly better survival than those unmarried. While, this difference was not significant in uninsured patients. This indicated that insurance and financial status might play the key roles in the survival difference between married and unmarried patients. Moreover, Asian patients had higher percentages of married than other races. Lots of studies showed that the prognosis for cancer was better for Asian than for Caucasian patients^22^,^23^,^24^,^25^. In our present study, we also found that race/ethnicity was an independent prognostic factor in the multivariable analysis. Comparing with Caucasian, there was a 22% decreased of cause-specific death in Asian patients. The survival difference between married and unmarried patients persisted in Caucasian, African-American and Asian patients. Except for the race/ethnicity issue, the earlier stage at presentation may also contribute to the better survival for married patients. Some studies showed that delayed diagnosis was one of the reasons for poor prognosis in unmarried patients^10^,^14^. Spouse might provide social supports and encourage the patients to seek medical treatments^26^,^27^. Though there is no information of chemotherapy in the SEER database, in our study, we found that more married patients received surgery with or without radiation than unmarried patients. Cancer patients may suffer from distress, depression and some other psychologic problems^28^. Married patients were reported to suffer less from distress, depression and anxiety than their unmarried counterparts, especially for male patients^29^,^30^. Partly because the spouse could share the emotional burden and provide supports to the patients^10^.

The comparison of clinicopathologic features among different marital status showed that patients in the widowed groups had more stage I and well or moderately differentiated tumors. Even with these good prognostic factors, patients in widowed group still ended up with the poorest 5-year CSS. The exact reasons need to be furtherly explored. From our present data, we found that widowed patients were older than patients in other groups. Comparing with the younger patients, elderly gastric cancer patients were reported to have a poorer survival^31^,^32^. Meanwhile, some studies showed that excess mortality after the death of a spouse was partly caused by stress^33^. Stress and depression might cause noncompliance to the medical treatment^34^,^35^.

Compared with female patients, male patients had a slightly better survival, the 5-year CSS was 28.8% vs 28.5%, P = 0.0285. Though the difference was statistically significant, the absolute difference was small. This P value may be caused by the big sample size.

Though marriage showed a significant protective effect in both male and female patients, male patients benefitted more from marriage than female patients did. The same phenomenon was reported by Aizer AA *et al.* in lung, colorectal, breast, pancreatic, prostate, liver/intrahepatic bile duct, non-Hodgkin lymphoma, head/neck, ovarian as well as esophageal cancer patients^10^. The author explained that maybe unmarried women received greater social support from their relatives, friends, or the community than unmarried men^10^. However, a systematic review and meta-analysis did not confirm the differential effect of marriage in men versus women^20^. As for the gender difference, there was one more point merited discussion. The ratio of male to female was about 2:1 in the whole population which was consistent to the previous reports^36^,^37^. However, the ratio of male to female reversed in the widowed subgroup which was about 1:2.28. One of the possible explanations was that the mortality rate was higher among widowers than widows^38^. The loss of social support and the inability to deal with stress might also explain why men suffer from bereavement more than women^33^. In the whole population, male patients had a slightly better survival than female patients. The association between marital status and gender was a complicated issue. Further data are required to support this hypothesis.

From the present knowledge, the benefits of marriage on survival might be mediated through social and psychologic support. It is therefore important for the physicians to screen for depression among gastric adenocarcinoma patients and consider closer observation as well as necessary psychologic support to these patients.

In 2015, Zhou RP *et al.* had reported that unmarried GC patients, especially widowed patients, were at a high risk of gastric cancer specific survival^15^. Though our present study is not the first study to analyze the survival disparity between married and unmarried gastric cancer patients, we not just replicated their main conclusion. Actually, we made a further analysis to seek out possible explanations for the survival difference. We used data to elucidate that insurance and financial status as well as race/ ethnicity may play important roles in the survival disparity. The another big difference between our present study and Zhou RP’s previous report is that we focus on the adenocarcinoma, which is the most common histologic subtype for gastric cancer. While Zhou RP *et al.* put carcinoid tumor/neuroendocrine, gastrointestinal stromal sarcoma and adenocarcinoma together.

Potential limitations of our study should be taken into consideration. Firstly, there may be some other factors that contribute to the survival disparity among different marital status patients, such as chemotherapy. However, data related to chemotherapy are not available in SEER database. Secondly, there is no data about the HP and EBV infection in the SEER database, we cannot make a comparison about infection among different marital status. Finally, the quality of marriage could not be assessed in this study. Since the support and accompany of spouse seem to be responsible for the good survival in married patients, the quality of marriage is an important issue.

In conclusion, we used the SEER database to evaluate the survival disparity of gastric adenocarcinoma patients with different marital status. Our data revealed that married patients had a survival advantage in both male and female patients. The relationship between marriage and survival can be explained hypothetically by psychosocial and socioeconomic factors. We recommend that every subject should have access to best available gastric cancer therapy.

## Methods

### Database

The SEER database is the largest publicly available cancer dataset. It is a population-based cancer registry across several disparate geographic regions. The SEER research data include cancer incidence and prevalence as well as demographic information tabulated by age, sex, race / ethnicity, year of diagnosis, marital status, insurance, Tumor-Node-Metastasis (TNM) stage and geographic region. The exact dataset we used for this analysis was SEER Program (www.seer.cancer.gov) Research Data (1973–2012), National Cancer Institute, DCCPS, Surveillance Research Program, Surveillance Systems Branch, released April 2015, based on the November 2014 submission, “Incidence-SEER 18 Regs Research Data + Hurricane Katrina Impacted Louisiana Cases, Nov 2014 Sub (1973–2012 varying)”.

### Outcome variables

Variable definitions information on age at diagnosis, sex, year of diagnosis, race/ethnicity, marital status, primary site, tumor grade and differentiation, histology, lymph node involvement, AJCC 6^th^ TNM stage, insurance status and overall survival were coded and available in SEER database (Appendix S1).

For the Race/Ethnicity, we reclassified the patients into 5 groups: “Caucasian”, “African American”, “Asian”, “Others” and “Unknown”.

Patients were classified as married and unmarried. Since the group of “Unmarried or domestic partner” is misleading and we removed this group of patients from analysis. Unmarried patients included single, separated/divorced and widowed.

Since the AJCC 7^th^ TNM staging system was released in 2010 and if we used this staging system, there would be no 5 year survival due to insufficient follow up and less patients, so we picked up the AJCC 6^th^ TNM staging systems. Meanwhile, since the AJCC 6^th^ TNM staging system was released in 2004, we restricted our study from 2004–2012. Patients were divided into “insured group” and “uninsured group” according to their insurance status.

### Patient Population

The study population was based on the SEER cancer registry. We restricted eligibility to adults (aged 18 years or older) who were diagnosed with gastric adenocarcinoma (also including mucinous adenocarcinoma and signet ring cell carcinoma) from 2004 to 2012. We excluded cases without follow-up records (survival time code of 0 months). Patients without record of marital status and TNM stage were also excluded.

### Statistical Methods

The patients’ demographic and tumor characteristics were summarized with descriptive statistics. Comparisons of categorical variables among different marital status were performed using the Chi square test, and continuous variables were compared using Student’s t test. The primary endpoint of this study was 5-year CSS, which was calculated from the date of diagnosis to the date of cancer specific death. Deaths attributed to gastric cancer were treated as events and deaths from other causes were treated as censored observations. Survival function estimation and comparison among different variables were performed using Kaplan–Meier estimates and the log-rank test. The independence of the prognostic effect of the marital status was evaluated by adjusting for other known factors including age at diagnosis and tumor stage. The multivariate Cox proportional hazard model was used to evaluate the hazard ratio (HR) and the 95% CI for all the known prognostic factors, including sex, age, marital status, location, race/ethnicity, time of diagnosis, TNM stage, lymph node removed, histology, grade, insurance status and therapy (Surgery with or without radiotherapy). All of statistical analyses were performed using the Intercooled Stata 13.0 (Stata Corporation, College Station, TX). Statistical significance was set at two-sided P < 0.05.

## Additional Information

**How to cite this article**: Qiu, M. *et al.* Impact of marital status on survival of gastric adenocarcinoma patients: Results from the Surveillance Epidemiology and End Results (SEER) Database. *Sci. Rep.* **6**, 21098; doi: 10.1038/srep21098 (2016).

## Supplementary Material

Supplementary Information

## Figures and Tables

**Table 1:** Basic characteristics.

	Married N (%) N = 17854	Unmarried N (%) N = 11220			*P* value
		Widowed N = 4353	Single N = 4139	Separated/Divorced N = 2728	
Sex					<0.001
Male	12,841(71.92)	1,325 (30.44)	2,564 (61.95)	1,554 56.96)	
Female	5,013 (28.08)	3,028 (69.56)	1,575 (38.05)	1,174 (43.04)	
Age (Mean ± SD)	64.5 ± 13.0	78.2 ± 9.4	58.3 ± 15.2	63.3 ± 11.9	<0.001
Race/Ethnicity					<0.001
Caucasian	12,829 (71.85)	2,971 (68.25)	2,654 (64.12)	1,909 (69.98)	
African-American	1,553 (8.70)	659 (15.14)	973 (23.51)	500 (18.33)	
Asian	3,137 (17.57)	647 (14.86)	413 (10.00)	257 (9.42)	
Others	309 (1.73)	74 (1.70)	97 (2.34)	56 (2.05)	
Unknown	26 (0.15)	2 (0.05)	2 (0.03)	6 (0.22)	
Insurance status					<0.001
Uninsured	401 (2.24)	35 (0.80)	276 (6.67)	100 (3.67)	
Insured	11,260 (63.07)	2,684 (61.66)	2,579 (62.31)	1,673 (61.33)	
Unknown	6193 (34.69)	1634 (37.54)	1284 (31.02)	955 (35.00)	
Location					<0.001
Cardia	6,096 (34.14)	940 (21.59)	1,198 (28.94)	900 (33.00)	
Fundus	636 (3.56)	167 (3.84)	135 (3.26)	110 (4.03)	
Body	1,509 (8.45)	422 (9.69)	346 (8.36)	247 (9.05)	
Antrum/Pylorus	3,991 (22.35)	1,326 (30.46)	1002 (24.21)	579 (21.22)	
Lesser/Greater curvature	2,168 (12.14)	564 (12.96)	484 (11.69)	330 (12.10)	
Others	3,454 (19.36)	934 (21.46)	974 (23.54)	562 (20.60)	
AJCC 6^th^ TNM stage					<0.001
I	4,976 (27.87)	1,601 (36.78)	982 (23.73)	728 (26.69)	
II	2,581 (14.46)	582 (13.37)	546 (13.18)	350 (12.83)	
III	2,521 (14.12)	552 (12.68)	564 (13.63)	379 (13.89)	
IV	7,776 (43.55)	1,618 (37.17)	2,047 (49.46)	1,271 (46.59)	
Lymph node removed					<0.001
1–3	746 (4.18)	215 (4.94)	177 (4.29)	110 (4.03)	
>3	8,779 (49.17)	1,827 (41.97)	1,707 (41.24)	1,217 (44.61)	
Unknown	8,329 (46.65)	2,311 (53.09)	2,261 (54.47)	1,401 (51.36)	
Histology subgroup					<0.001
Signet Ring cell	3,910 (21.90)	724 (16.63)	1,063 (25.68)	597 (21.88)	
Mucinous adenocarcinoma	420 (2.35)	114 (2.62)	120 (2.90)	63 (2.31)	
Adenocarcinoma	13,524 (75.75)	3,515 (80.75)	2,965 (71.42)	2,068 (75.81)	
Grade					<0.001
Well differentiated	640 (3.58)	212 (4.87)	109 (2.63)	104 (3.81)	
Moderately differentiated	4,067 (22.78)	1,137 (26.12)	884 (21.36)	620 (22.73)	
Poorly differentiated	10,512 (58.88)	2,349 (53.96)	2,480 (59.92)	1,552 (56.89)	
Undifferentiated	351 (1.97)	76 (1.75)	67 (1.62)	46 (1.69)	
Unknown	2,284 (12.79)	579 (13.30)	599 (14.47)	406 (14.88)	
Therapy (Surgery ± radiotherapy)					<0.001
None	5,794 (32.45)	1,548 (35.56)	1,352 (32.66)	967 (35.45)	
Therapy	10,111 (56.63)	2,314 (53.16)	2,221 (53.66)	1,449 (53.12)	
Unknown	1,949 (10.92)	491 (11.28)	566 (13.67)	312 (11.43)	

SD: Standard deviation.

NOS: Not otherwise specified.

AJCC: American Joint Committee on Cancer.

TNM: Tumor-Node-Metastasis.

**Table 2:** Univariate analysis of cause specific survival.

	Median CSS	5-year CSS (95% CI)	Univariate analysis	P value
			Log rank	
Sex			4.8	0.0285
Male	16.48	28.8 (28.0–29.6)		
Female	15.35	28.5 (27.5–29.6)		
Age			10.6	0.0011
<67	16.41	28.7 (27.5–29.3)		
>66	15.77	29.1 (28.1–30.0)		
Marital status			149.37	<0.001
Married	17.87	30.6 (29.7–31.4)		
Widowed	13.21	25.3 (24.2–27.4)		
Single	13.29	25.4 (23.7–27.1)		
Separate/Divorced	14.69	26.2 (24.2–28.2)		
Race/Ethnicity			293.33	<0.001
Caucasian	15.10	26.6 (25.9–27.4)		
African-American	14.50	26.7 (25.0–28.5)		
Asian	28.00	40.1 (38.3–41.8)		
Others	17.53	34.6 (25.5–43.9)		
Insurance status			28.0	<0.001
Uninsured	11.43	21.4 (17.3–25.8)		
Insured	16.95	29.9 (28.9–30.8)		
Location			999.05	<0.001
Cardia	16.38	25.4 (24.2–26.5)		
Fundus	13.51	24.0 (20.8–27.3)		
Body	16.77	30.9 (28.6–33.1)		
Antrum/Pylorus	23.10	37.0 (35.6–38.3)		
Lesser/Greater curvature	26.32	39.0 (37.0–40.9)		
Others	9.22	17.6 (16.4–18.8)		
AJCC 6^th^ TNM stage			9814.31	<0.001
I	–	61.9 (60.6–63.2)		
II	36.82	40.9 (39.0–42.9)		
III	19.47	23.3 (21.6–25.0)		
IV	7.11	4.8 (4.3–5.3)		
Lymph node removed			34.11	<0.001
1–3	30.43	39.5 (36.3–42.7)		
>3	44.13	45.7 (44.6–46.7)		
Histology subgroup			173.40	<0.001
Signet Ring cell	12.73	21.6 (20.3–22.8)		
Mucinous adenocarcinoma	17.59	29.4 (25.5–33.4)		
Adenocarcinoma	17.45	30.8 (30.0–31.5)		
Grade			682.88	<0.001
Well differentiated	–	58.7 (55.1–62.2)		
Moderately differentiated	27.71	39.4 (37.9–40.8)		
Poorly differentiated	14.55	24.9 (24.1–25.7)		
Undifferentiated	14.20	22.4 (18.1–26.9)		
Therapy (Surgery ± radiotherapy)			9457.52	<0.001
Therapy	44.02	45.7 (44.8–46.6)		
None	6.24	4.4 (3.8–5.0)		
Unknown	10.37	0		

CSS: Cause specific survival.

CI: Confidence interval.

AJCC: American Joint Committee on Cancer.

TNM: Tumor-Node-Metastasis.

**Table 3:** Multivariate analysis of survival.

	Hazard ratio (95% confidence interval)	*P* value
Sex		
Male	Reference	
Female	0.96 (0.89–1.04)	0.313
Age		
<67	Reference	
>66	1.45 (1.35–1.56)	<0.001
Marital status		
Married	Reference	
Unmarried	1.09 (1.01–1.17)	0.027
Race/Ethnicity		
Caucasian	Reference	
African-American	1.03 (0.93–1.14)	0.580
Asian	0.78 (0.71–0.86)	<0.001
Others	0.96 (0.73–1.26)	0.759
Insurance status		
Insured	Reference	
Uninsured	1.08 (0.88–1.31)	0.472
Location		
Cardia	Reference	
Fundus	0.90 (0.73–1.10)	0.297
Body	0.81 (0.70–0.93)	0.002
Antrum/Pylorus	0.83 (0.75–0.91)	<0.001
Lesser/greater curvature	0.77 (0.69–0.87)	<0.001
Others	0.97 (0.87–1.09)	0.641
AJCC 6^th^ TNM stage		
I	Reference	
II	2.58 (2.29–2.90)	<0.001
III	4.43 (3.96–4.95)	<0.001
IV	7.75 (6.94–8.67)	<0.001
Lymph node removed		
1–3	Reference	
>3	0.64 (0.56–0.72)	<0.001
Histology subgroup		
Adenocarcinoma	Reference	
Signet Ring cell	1.14 (1.04–1.24)	0.003
Mucinous adenocarcinoma	1.06 (0.87–1.30)	0.563
Grade		
Well differentiated	Reference	
Moderately differentiated	1.39 (1.08–1.80)	0.012
Poorly differentiated	1.95 (1.52–2.50)	<0.001
Undifferentiated	2.18 (1.60–2.98)	<0.001
Therapy (Surgery ± radiotherapy)		
Therapy	Reference	
None	2.06 (1.92–2.23)	<0.001
Unknown	1.51 (1.43–1.59)	<0.001

AJCC: American Joint Committee on Cancer.

TNM: Tumor-Node-Metastasis.
