# Supplementary material for: Impact of marital status on survival of gastric adenocarcinoma patients: Results from the Surveillance Epidemiology and End Results (SEER) Database
Source: Sci Rep. 2016 Feb 15;6:21098. doi: 10.1038/srep21098 (PMC4753440; doi:10.1038/srep21098)
Supplement: Supplementary Information [file srep21098-s1.doc]

**Impact of marital status on survival of gastric adenocarcinoma patients: Results from the Surveillance Epidemiology and End Results (SEER) Database.**

**Miaozhen Qiu, Dajun Yang, Ruihua Xu**

**Appendix S1** Program codes for Surveillance, Epidemiology, and End Results (SEER) database queries

For the Race/Ethnicity, we reclassified the patients into 5 groups: “Caucasian” (Race/Ethnicity code, 1), “African American” (Race/Ethnicity code, 2), “Asian” (Race/Ethnicity code, 4-6, 8, 10-17 and 96), “Others” (The rest code, except for the code for unknown) and “Unknown” (Race/Ethnicity code, 99).

Patients were classified as married and unmarried. Since the group of “Unmarried or domestic partner” is misleading and we removed this group of patients from analysis. Unmarried patients included single, separated/divorced (“Separated” and “Divorced”) and widowed.

The primary site was defined by the following International Classification of Diseases for Oncology (ICD-O-2) codes: C16.0-C16.9. Cardia, (C16.0), fundus (C16.1), body (C16.2), antrum (C16.3), pylorus (C16.4), lesser curvature (C16.5), greater curvature (C16.6), overlapping lesion (C16.7) and stomach, NOS (C16.9). We combined the antrum and pylorus into one group, lesser and greater curvature into one group. Overlapping lesion and stomach, NOS were classified as others.

Grade and differentiated was defined by the following ICD-O-2 codes; well differentiated (Code 1), moderate differentiated (Code 2), poorly differentiated (Code 3) and undifferentiated (Code 4).

Histological types were defined by the following ICD-O-3 codes: 8140 to 8147, 8210 to 8211, 8220 to 8221, and 8260 to 8263 for adenocarcinoma, 8480 and 8481 for mucinous adenocarcinoma, and 8490 for Signet ring cell carcinoma.

The number of lymph nodes resected for patients who received surgical resection was classified as 1-3 lymph nodes and more than three lymph nodes.

Since the AJCC 7^th^ TNM staging system was released in 2010 and if we used this staging system, there would be no 5 year survival due to insufficient follow up and less patients, so we picked up the AJCC 6^th^ TNM staging systems. Meanwhile, since the AJCC 6^th^ TNM staging system was released in 2004, we restricted our study from 2004-2012.

For the insurance status, individuals in the “Any Medicaid”, “Insured” and “Insured/No specifics” groups were clustered together as “Insured group”. Patients were therefore divided into “insured group” and “uninsured group”.
